# Comparison of Two Generations of Self-Expandable Transcatheter Heart Valves in Nine Surgical Valves: An In Vitro Study

**DOI:** 10.3390/jcdd11080244

**Published:** 2024-08-08

**Authors:** Najla Sadat, Michael Scharfschwerdt, Stephan Ensminger, Buntaro Fujita

**Affiliations:** 1 Department of Cardiac and Thoracic Vascular Surgery, University Medical Center Schleswig-Holstein, Campus Luebeck, 23538 Lubeck, Germany; 2 German Center for Cardiovascular Research (DZHK), Partner Site Hamburg/Kiel/Luebeck, 20246 Hamburg, Germany

**Keywords:** transcatheter heart valve, TAVI, valve-in-valve, surgical aortic valve, bioprosthetic valve, Evolut R, Evolut PRO, pulse duplicator

## Abstract

(1) Background: This study aimed to analyse the hydrodynamic performance of two generations of self-expanding transcatheter heart valves (THV) as a valve-in-valve (ViV) in different surgical aortic valve (SAV) models under standardised conditions. The nitinol-based Evolut R valve is frequently used in ViV procedures. It is unclear whether its successor, the Evolut PRO, is superior in ViV procedures, particularly considering the previously implanted SAV model. (2) Methods: Evolut^TM^ R 26 mm and Evolut^TM^ PRO 26 mm prostheses were implanted in nine 21 mm labelled size SAV models (Hancock^®^ II, Mosaic^®^ Ultra^TM^, Epic^TM^ Supra, Trifecta^TM^ GT, Perimount^®^, Perimount^®^ Magna Ease, Avalus^TM^, Intuity^TM^, Freestyle^®^) to analyse their hydrodynamic performance under defined circulatory conditions in a pulse duplicator. (3) Results: Both THVs presented with the lowest effective orifice area (EOA) and highest mean pressure gradient (MPG) inside Hancock^®^ II, whereas THVs in Intuity showed the highest EOA and lowest MPG. Evolut R and Evolut PRO showed significant hydrodynamic differences depending on the SAV. Both THVs performed similarly in porcine valves. Although the Evolut R performed better than Evolut PRO in stented bovine SAVs, the Evolut PRO was superior inside the Intuity. Further, the SAV model design markedly influenced the TAV’s geometric orifice area and pin-wheeling index. (4) Conclusions: These findings show that the Evolut R and Evolut PRO perform differently depending on the previously implanted SAV model. THV selection for treatment of a specific SAV model should consider these results.

## 1. Introduction

Transcatheter aortic valve implantation (TAVI) is a well-performed procedure for high-risk and older patients with aortic valve stenosis. It has become an established treatment option for failed surgical bioprosthetic valves [1,2]. Compared with redo surgical aortic valve replacement (SAVR), valve-in-valve (ViV) procedures can be performed less invasively and improve the hemodynamics of the degenerated bioprostheses [3]. Currently, two transcatheter heart valve (THV) models are approved for use in ViV procedures: the balloon-expandable SAPIEN valve and the self-expanding Evolut prosthesis [3]. Both valve models have undergone significant improvements in valve design and have improved outcomes for the patients [4,5,6,7]. However, these modifications were primarily made for the treatment of native aortic valve stenosis. Seiffert et al. showed that the newest generation balloon-expandable THV (SAPIEN3) was not superior to its predecessor model (SAPIEN XT) in terms of hemodynamic outcomes for the treatment of failed bioprosthetic valves [8]. It is currently unclear how the newest generation self-expanding model Evolut PRO compares to its predecessor Evolut R when implanted inside a degenerated surgical valve. Moreover, the functional results of a ViV procedure largely depend on the design of the degenerated bioprosthesis and the implanted THV. Therefore, the Evolut PRO and R may behave differently depending on the surgical valve model.

This study aimed to compare the functional performance of the Evolut PRO implanted inside frequently used surgical valves with the Evolut R using a standardised in vitro setting.

## 2. Materials and Methods

### 2.1. Aortic Valve Bioprostheses

**Figure 1:**
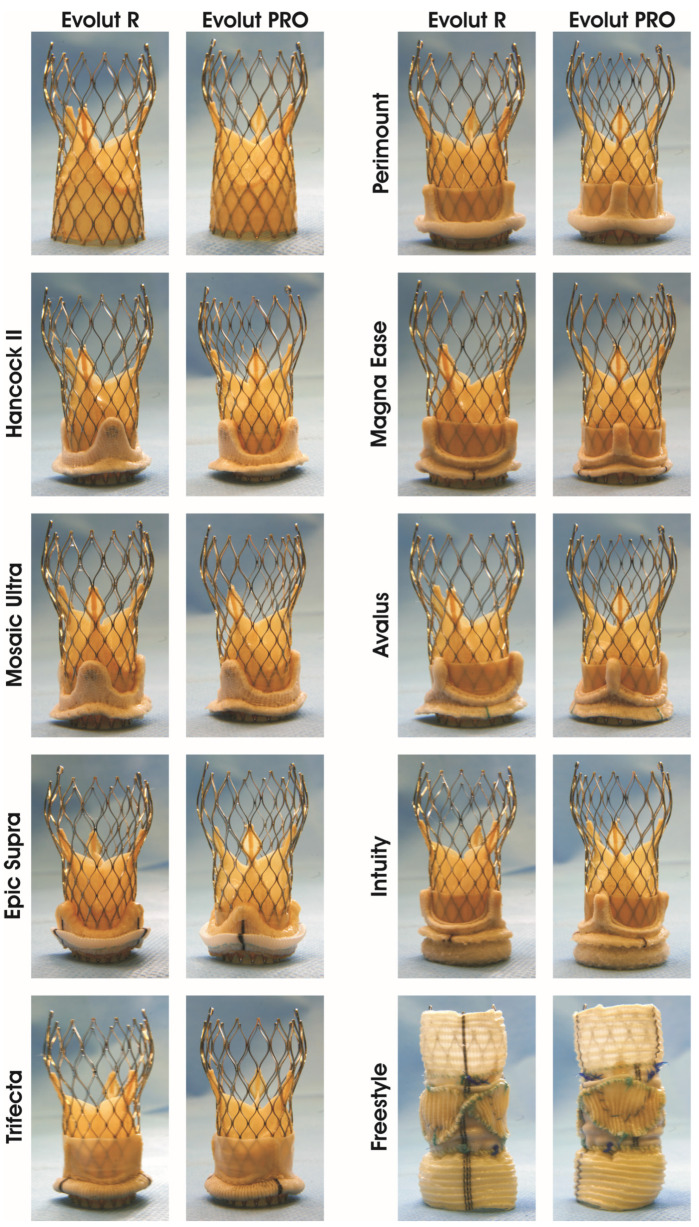
Images of Evolut R and Evolut PRO as valve-in-valve inside nine surgical aortic bioprostheses models. Stented porcine surgical aortic surgical bioprostheses (Hancock II, Mosaic Ultra and Epic Supra), stented bovine bioprostheses (Trifecta, Perimount, Magna Ease, Avalus and Intuity) and stentless porcine bioprosthesis (Freestyle).

We chose nine surgical aortic valves (SAVs) with a labelled size of 21 mm, which are currently commercially available and have been frequently implanted in the past: the Hancock^®^ II, Avalus^TM^, Mosaic^TM^ Ultra, Freestyle^®^ (Medtronic, Minneapolis, MI, USA); Perimount^®^, Perimount^®^ Magna Ease, Intuity^®^ (Edwards Life-Sciences, Irvine, CA, USA), Trifecta^TM^ GT (St. Jude Medical, Saint Paul, MI, USA) and Epic^TM^ Supra (Abbott, Chicago, IL, USA) (Figure 1). The THVs used in this study were the self-expanding Evolut^TM^ R 26 mm and its successor Evolut^TM^ PRO 26 mm (Medtronic, MI, MA, USA). 

**Figure 2:**
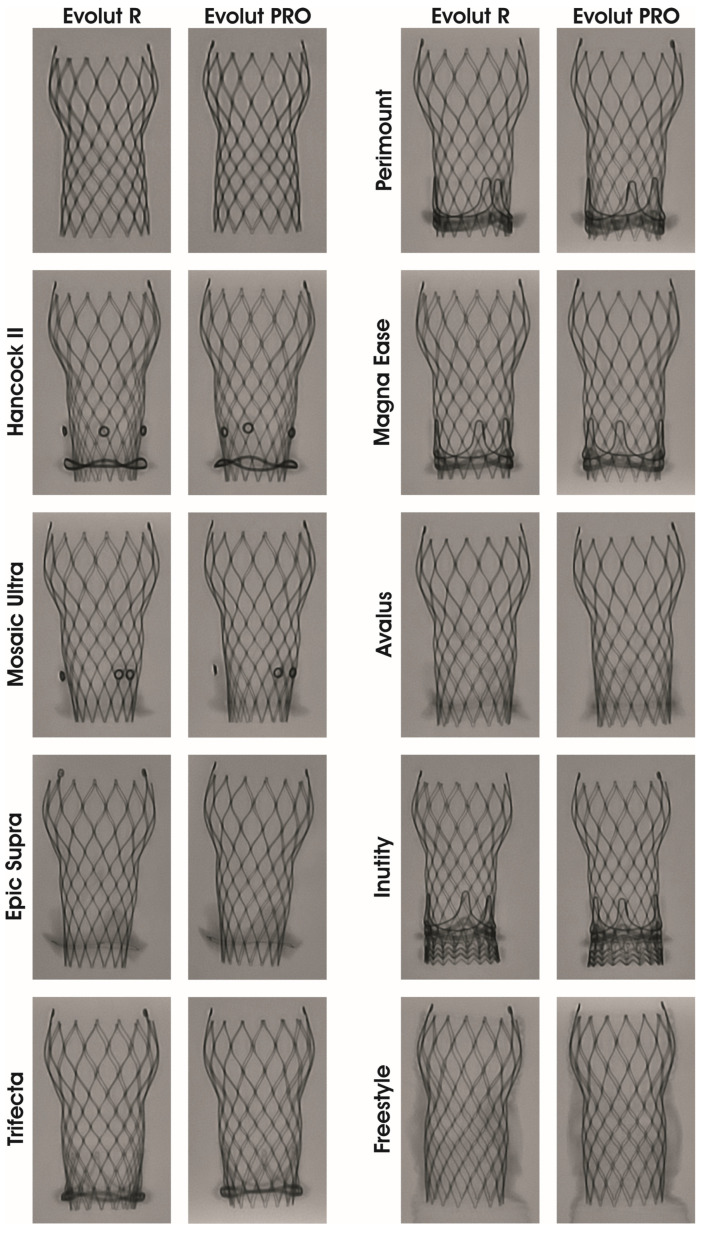
Fluoroscopic images of Evolut R and Evolut PRO as valve-in-valve inside nine surgical aortic bioprostheses models. Stented porcine surgical aortic surgical bioprostheses (Hancock II, Mosaic Ultra and Epic Supra), stented bovine bioprostheses (Trifecta, Perimount, Magna Ease, Avalus and Intuity) and stentless porcine bioprostheses (Freestyle).

The two THVs were crimped using the manufacturer’s original crimping set. Deployment inside the SAV was performed under direct vision. Fluoroscopic images were obtained to evaluate the implantation depth and expansion of the THV inside the SAVs. The THV was positioned according to the valve-in-valve app [9], as shown in Figure 1 and Figure 2. The implantation depth of the THV was 4 mm from the margin of the SAV ring to the tip of the Evolut (Figure 2).

### 2.2. Hydrodynamic Measurement

**Figure 3:**
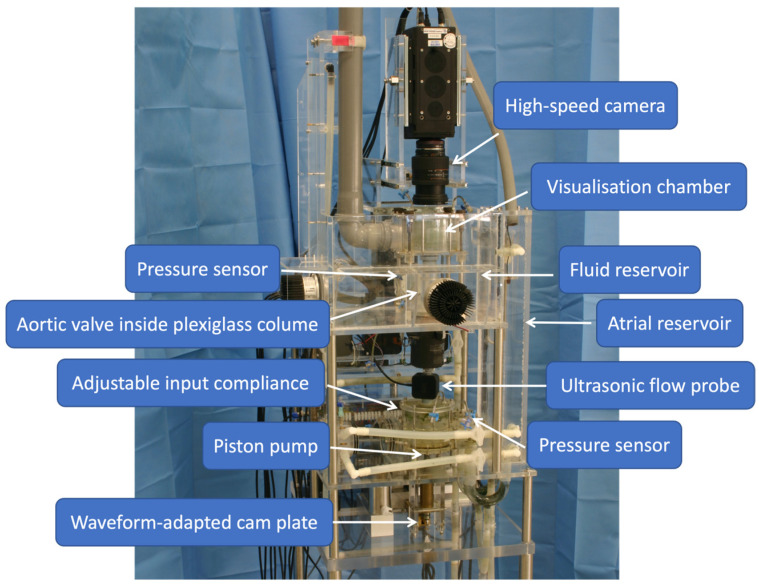
A custom-made pulse duplicator system according to ISO 5840 regulations for heart valve testing [10,11].

THV performance was assessed using the pulse duplicator system described in detail by Schwarfschwerdt et al. [10]. The pulse duplicator mimics the left ventricular function using a piston pump driven by waveform-adapted cam plates [10]. This way, stroke volume and beating rate can be adjusted to generate different cardiac outputs through the aortic valve compartment. Saline at 37 °C is used as test fluid. Further elements of the pulse duplicator are an atrial reservoir, a fluid reservoir, adjustable aortic compliance, pressure sensors, an ultrasonic flow probe and a high-speed camera above a visualisation chamber to enable a view of the tested heart valves (Figure 3). The system was designed to meet ISO 5840 standards for testing heart valve prostheses [10,11].

In this study, valves were perfused at a constant heart rate of 64/min and four different circulatory conditions with stroke volumes of 55 mL, 70 mL, 90 mL and 105 mL. One measurement included ten cycles. Five measurements were performed per ViV combination at each condition (total measurements = 360). The mean pressure gradient (MPG) in mmHg and effective orifice area (EOA) in cm^2^ were obtained from ISO requirements [11]. The pressure gradient was measured at 6.5 cm below and 4 cm above the valve with a pressure transducer (Envec Ceracore M capacitive pressure transducers, Endress and Hauser, Maulburg, Germany). The flow through the valve was measured with an ultrasonic flowmeter (HT207, Transonic Systems Inc., Ithaca, NY, USA) [10,11]. We used a trigger signal to synchronise all values with high-speed camera recordings [10]. The EOA was calculated using a simplified Bernoulli equation, where Q_RMS_ is the forward flow and Δp_mean_ is the MPG:

$$EOA=\frac{Q_{RMS}}{51.6\sqrt{∆p_{mean}}}$$

THV leaflet kinematics were recorded at 500 frames/s with a high-speed video camera (Motionpro Y-1000, Imaging Solutions GmbH, Eningen, Germany) integrated into the pulse duplicator system. The following parameters were obtained at a frequency of 64/min and a stroke volume of 70 mL.

**Figure 4:**
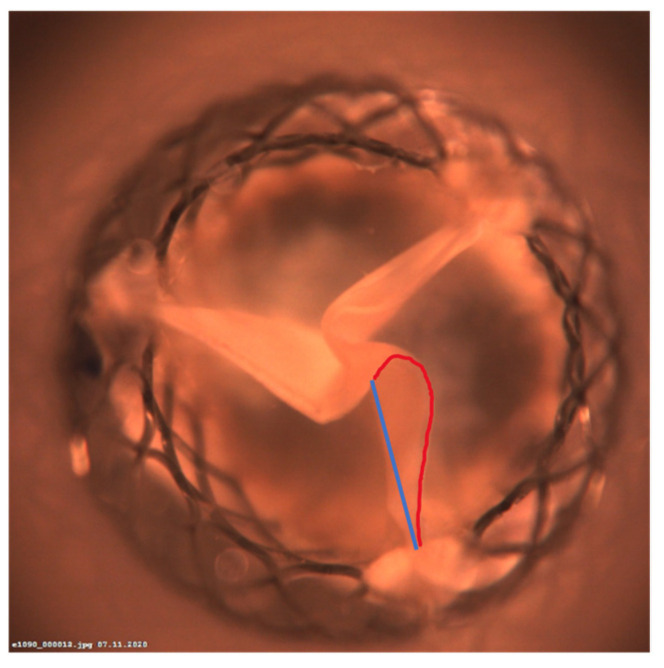
The PWI is calculated from the straight distance (L_ideal_) between the valve frame and the coaptation centre (marked in blue) and the traced length (L_actual_) of the leaflet’s free edge between these two points (marked in red).

(1) The valves’ maximal geometrical orifice area (GOA) was determined in cm^2^ at each frame by tracing the edges of the opening area (the black area inside the valve).
(2) The minimal internal diameter (MID) to evaluate the expansion of the THV is calculated using the following formula, where the GOA_max_ represents the maximal opening of the THV, and the MID considers the kinematic of all three leaflets:
  $$MID=\sqrt{\frac{{GOA}_{max}}{\pi }}\times 2.$$
(3) The pin-wheeling index (PWI) to quantify the degree of leaflet folding is calculated using the following equation, as illustrated in Figure 4 [12]:
  $$PWI\;(\%)=\frac{L_{actual}-L_{ideal}}{L_{ideal}}\times 100$$

### 2.3. Statistical Analysis

For statistical evaluation, R version 4.1.0 (R Core Team. R: A Language and Environment for Statistical Computing. R Foundation for Statistical Computing, Vienna, Austria, 2021) was used. Quantitative data are summarised as the median (25th–75th percentile) and graphically presented as Tukey boxplots. The Mann–Whitney U test was performed to compare two independent groups, and the Kruskal–Wallis test was used to test more than two groups. The Wilcoxon rank sum test with Bonferroni–Holm correction was performed for pairwise testing of independent groups. All tests were two-sided; a *p* < 0.05 was considered statistically significant.

## 3. Results

### 3.1. Hydrodynamic Comparison of Evolut R versus Evolut PRO as ViV

**Figure 5:**
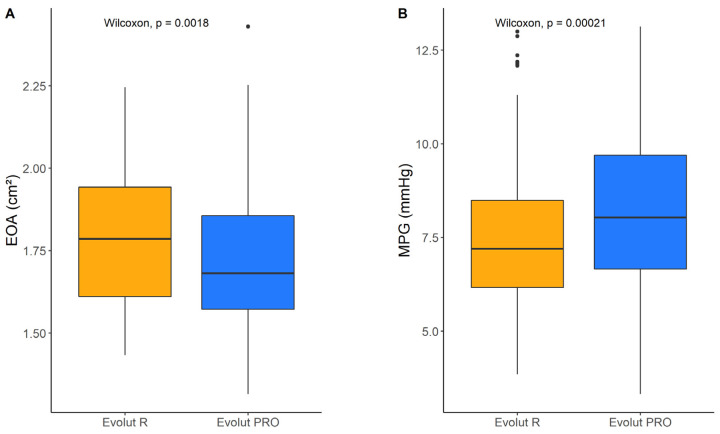
Hydrodynamic data I: (**A**) effective orifice area (EOA) and (**B**) mean pressure gradient (MPG) of Evolut R vs. Evolut PRO as valve-in-valve. Data are presented as boxplots (median and 25th–75th interquartile; dots indicate outliers) of 180 measurements for each THV model (5 per circulatory condition 50 mL, 70 mL, 90 mL, and 105 mL per 9 surgical valve models).

First, we compared the hydrodynamics of the two THVs across all SAVs. The Evolut R presented with a significantly higher EOA (1.79 [1.61–1.94] cm^2^ vs. 1.68 [1.57–1.86] cm^2^; *p* = 0.0018) and significantly lower MPG (7.20 [6.16–8.49] mmHg vs. 8.03 [6.66–9.70] mmHg; *p* = 0.00021) (Figure 5).

**Figure 6:**
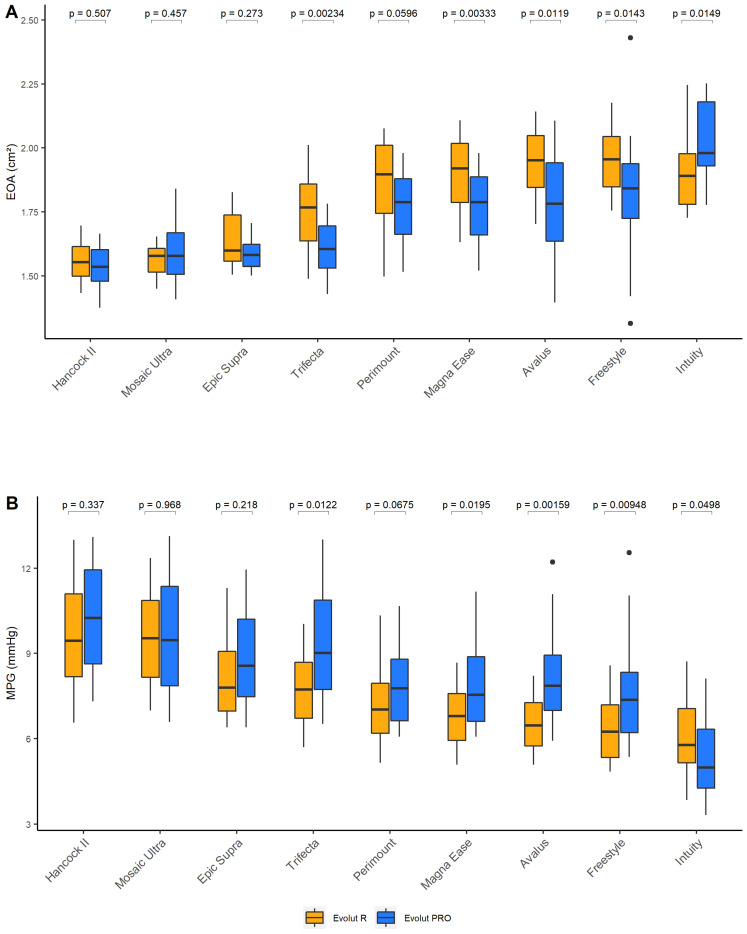
Hydrodynamic data II: (**A**) effective orifice area (EOA) and (**B**) mean pressure gradient (MPG) of Evolut R vs. Evolut PRO inside specific surgical valve models. Data are presented as boxplots (median and 25th–75th interquartile; dots indicate outliers) of 20 measurements per valve combination (5 per circulatory condition 50 mL, 70 mL, 90 mL, and 105 mL).

Next, we investigated the performance of the two THVs inside each SAV. Tables S1, S2 and Figure 5 summarise the hydrodynamic parameters EOA and MPG for the Evolut R and Evolut PRO implanted in the various SAVs. Regarding EOA, the Evolut R and Evolut PRO achieved similar values inside the Hancock II (1.55 [1.50–1.61] cm^2^ vs. 1.53 [1.48–1.60] cm^2^; *p* = 0.507), Mosaic Ultra (1.58 [1.51–1.61] cm^2^ vs. 1.58 [1.51–1.67] cm^2^; *p* = 0.457) and Epic Supra (1.60 [1.56–1.74] cm^2^ vs. 1.58 [1.54–1.62] cm^2^; *p* = 0.273) (Figure 5A). The Evolut R was superior to Evolut PRO inside the Trifecta (1.77 [1.64–1.86] cm^2^ vs. 1.60 [1.53–1.70] cm^2^; *p* = 0.002), Perimount (1.90 [1.74–2.01] cm^2^ vs. 1.79 [1.66–1.88] cm^2^; *p* = 0.06), Magna Ease (1.92 [1.79–2.02] cm^2^ vs. 1.79 [1.66–1.89] cm^2^; *p* = 0.003), Avalus (1.95 [1.85–2.05] cm^2^ vs. 1.78 [1.64–1.94] cm^2^; *p* = 0.012) and Freestyle (1.96 [1.85–2.04] cm^2^ vs. 1.84 [1.72–1.94] cm^2^; *p* = 0.014). The Evolut R was inferior to Evolut PRO inside the Intuity valve (1.89 [1.78–1.98] cm^2^ vs. 1.98 [1.93–2.18] cm^2^; *p* = 0.015) (Figure 6A). A similar pattern was seen regarding MPG, whereby the Evolut R and PRO performed similarly inside porcine valves; the Evolut R was superior inside stented bovine valves, and the Evolut PRO was superior inside the Intuity valve (Figure 6B).

### 3.2. Hydrodynamic Performance of Evolut R Regarding SAV Model

The EOA and MPG of Evolut R differed significantly in stented porcine valves (Hancock II, Mosaic Ultra and Epic Supra) in comparison to Evolut R in Freestyle and bovine pericardial valves (Table S1). Evolut R presented the highest MPG and lowest EOA in stented porcine valves (Table S1). The EOA and MPG of Evolut R were not significantly different amongst the porcine stented valves. Also, the EOA and MPG of Evolut R were not significantly different within the bovine pericardial group with internally mounted leaflets (Perimount, Magna Ease, Avalus and Intuity).

### 3.3. Hydrodynamic Performance of Evolut PRO Regarding the SAV Model

Evolut PRO as ViV presented significantly different EOA and MPG values depending on the surgical valve model (Table S2). The highest MPG and lowest EOA of Evolut PRO were observed in Hancock II, Mosaic Ultra and Epic Supra, whereas Evolut PRO performed significantly better inside Perimount, Avalus, Magna Ease, Intuity and Freestyle.

### 3.4. Leaflet Kinematic of Evolut R and Evolut PRO

**Figure 7:**
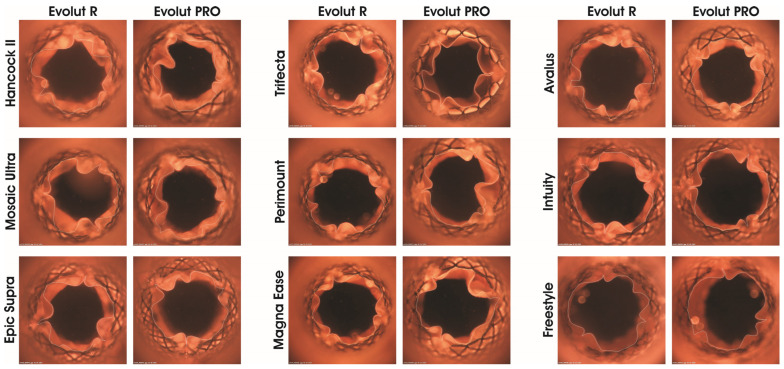
Images of Evolut R and PRO as valve-in-valve inside surgical aortic valve models at maximal opening during perfusion (pulse duplicator testing). The black area with the THVs represents the maximal geometric orifice area. Measurement was performed at a frequency of 64/min and a stroke volume of 70 mL.

The maximal GOA and MID were analysed to characterise THV expansion further. Both THVs demonstrated differences concerning the SAV in which they were implanted (Figure 7 and Table S3). The Evolut R and PRO showed the lowest GOA in Hancock II (GOA_max_~1.0 cm^2^), whereas the highest GOA was seen in Intuity (GOA_max_~2.0 cm^2^) (Table S3). Additionally, the lowest MID of both THVs was observed inside Hancock II (MID~16 cm^2^) and the highest was observed inside Intuity (MID~16 cm^2^) (Table S3).

**Figure 8:**
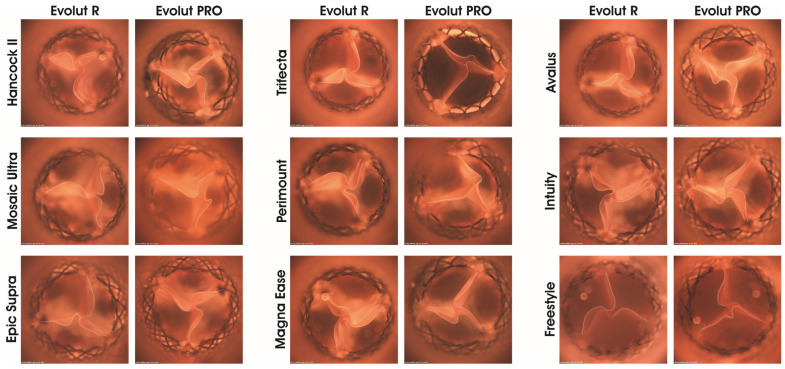
Images of Evolut R and PRO as valve-in-valve inside surgical aortic valve models at closed state during perfusion (pulse duplicator testing). The white lines represent the leaflet folding of the THV as valve-in-valve. Measurement was performed at a frequency of 64/min and a stroke volume of 70 mL.

The PWI_mean_ of the THVs were higher inside stented porcine valves (PWI_mean_ 19–27%) versus inside the bovine valves (PWI_mean_ 13–18%) and inside the Freestyle (PWI_mean_ 14%) (Table S3). Both THVs showed the highest PWI_mean_ in Hancock II and the lowest in Intuity. During complete leaflet closing, the stented porcine SAV (Hancock II, Mosaic Ultra and Epic Supra) caused extended leaflet folding of the THV in comparison to the stented bovine SAV (Trifecta, Perimount, Magna Ease and Intuity) and Freestyle (Figure 8). Further, the SAV model affected the leaflet kinematics of Evolut R and Evolut PRO. The leaflet kinematics of Evolut showed higher motion (“leaflet fluttering”) inside porcine SAVs than the Evolut leaflets inside the bovine SAVs (Videos S1 and S2).

## 4. Discussion

Our in vitro study systematically investigated the hydrodynamic performance of two generations of self-expanding THVs as valve-in-valve procedures in nine different SAV prosthesis models under standardised conditions. The key findings of this study are as follows:

(1) Generally, Evolut R performed significantly better than Evolut PRO in terms of EOA and MPG.
(2) The design of the SAV in which the two THVs were deployed significantly influenced the performance of both THVs. Both valves performed similarly in porcine valves; Evolut R was superior to Evolut PRO in stented bovine valves and Evolut PRO performed better within the Intuity valve.

### 4.1. Comparison of Evolut R vs. Evolut PRO

Evolut R generally achieved a larger EOA and lower MPG than Evolut PRO (Figure 3). Because both THVs have identical stent dimensions (inflow 26 mm, waist 22 mm and outflow 32 mm), the most reasonable explanation for this finding is the presence of an outer skirt at the inflow portion of Evolut PRO (13 mm in height), which impairs expansion of the THV stent [13]. The addition of the skirt was intended to reduce paravalvular leakage after TAVI in native aortic stenosis. However, paravalvular leakage is rarely a problem after ViV procedures. Therefore, this feature may not be associated with improving ViV procedures. A similar evolution was seen in the balloon-expandable SAPIEN XT (XT) and its successor, the SAPIEN3 valve (S3) [8]. In a clinical study, a comparison of the XT and S3 showed that transvalvular gradients were slightly (but not statistically significant) higher with the S3 compared with the XT, indicating that the addition of an outer skirt may lead to impaired hemodynamics of the THV within the surgical valve [8]. Considering the low risk of paravalvular leak (PVL), utilising the Evolut PRO or S3 for ViV procedures may not be sensible. However, the observed differences were minimal (~1–2 mmHg) and may not be relevant in the clinical setting.

On the other hand, a past study demonstrated that an increase in MPG by 5 mmHg is associated with a significantly increased risk of late (>10 years) heart failure after SAVR [14]. Therefore, in borderline cases, such differences in performance may become relevant. Regarding MPG and EOA, we conclude that the Evolut PRO cannot generally be recommended instead of the Evolut R for use in ViV procedures without further consideration.

### 4.2. Impact of the SAV Model on the THV as Valve-in-Valve

Interestingly, though, Evolut R and PRO performed differently depending on the model of the previously implanted SAV. Evolut R performed similarly to PRO in porcine valves, whereas Evolut R was superior to PRO in most stented bovine pericardial valves, and Evolut PRO outperformed Evolut R inside Intuity (Figure 6). As mentioned earlier, the outer skirt is the only difference between the Evolut R and PRO. In addition, the Evolut R and PRO were both implanted at the same implantation depths. The outer skirt, therefore, represents the most likely source of this finding. Porcine valves have relatively thick external sewing ring diameters, small internal stent diameters and even smaller true internal diameters [15]. These features contribute to impaired THV expansion. In addition, the cusps of porcine valves are made of actual aortic valve cusps, which show radial folding. The implantation of a THV inside porcine valves can result in the capture of a fold between the THV stent and the inner stent frame of the SAV.

In contrast, implantation of a THV inside a bovine pericardial valve leads to outward displacement of the SAV cusps, forming a tube [16]. These aspects may explain the different hydrodynamic results observed in the two investigated THVs inside the different SAV models. Particularly, the outer skirt of the Evolut PRO can play an important role as ViV regarding regurgitation as the skirt can functionally seal the space between the surgical valve and a THV.

However, further investigations are required to systematically analyse the outer skirt’s impact on ViV’s regurgitation as our study does not include this parameter. However, the differences in THV performance depending on the SAV were minor. Whether these differences are of clinical relevance remains to be investigated.

Our study also showed that increased gradients are associated with increased PWIs (pin-wheeling index). PWI quantifies the degree of under expansion, which was very high inside stented porcine valves (PWI_mean_ 19–27%). On the one hand, the true internal diameter of stented porcine valves (15–16 mm) is smaller than the true internal diameter of bovine pericardium SAV (17–21 mm) [15]. On the other hand, we also used 26 mm THVs instead of 23 mm THVs for all SAV models. The recommendation for a 21 mm labelled size surgical valve is a 23 mm labelled size THV, according to the valve-in-valve-app [9]. In the clinical setting, oversizing for ViV is controversial and disputed, and more studies are needed in this field to find the limitations of oversizing and the cases where an oversized THV can be used because of the surgical valve design. But as the true internal diameters of the surgical valves differ with a significant difference in their functional performance, we recognised a need to investigate the hydrodynamic performance of the oversized—26 mm–Evolut THVs inside small—21 mm—surgical aortic valves [15]. In vitro testing enables us to investigate these borderline questions, which cannot be evaluated in a clinical setting because of ethical reasons and possible disadvantages for patients. As expected, both Evolut THVs inside the stented porcine valves with small true internal diameters (Hancock II, Mosaic Ultra and Epic Supra) showed the most notable hydrodynamic impairment in our measurements (Tables S1 and S2). Additionally, the true internal diameter difference and one size larger THV may maintain high PWIs in our study. Apart from impaired hemodynamics, a high PWI has been suggested to be associated with impaired valve durability. Therefore, further investigation is needed to clarify the impact of the ViV combination on the durability of the THV. Our results further underline the importance of selecting the right SAV for later ViV therapy during the primary procedure, as this is the fundamental base for a good long-term outcome. Finally, to optimise outcomes of ViV-TAVI in small-sized SAV, pre- and post-procedural strategies such as balloon valve fracturing or remodelling of SAV and very precise positioning of the TAV within the SAV are useful strategies to optimise hemodynamic outcomes after ViV therapy [17,18,19]. Here, the SAV models and the used frame material are of utmost importance as some SAVs can be fractured and others can be remodelled to enlarge the SAV frame and improve the THV’s functional performance as ViV. Here, the degree of functional improvement gained depends on the previously implanted SAV model [18,20,21]. However, an enlargement of the SAV frame for ViV implantation allows better THV expansion, resulting in lower PWI [22]. Additionally, the implantation depth of the Evolut THV inside the SAV depends on the SAV model. Here, we reported that in a previous study, deeper implantation than 4 mm of the Evolut inside Magna Ease, Trifecta and Hancock II resulted in worse hydrodynamics and higher PWIs. Conversely, too high implantation (>3 mm) may also cause suboptimal functioning due to unsteady fixation and the tipping of the THV within the SAV [19].

The current study’s data are a valuable addition and, if combined with these strategies, may allow further optimisation of hemodynamics after ViV procedures in small bioprosthetic valves.

### 4.3. Study Limitations

We performed our measurements with bioprostheses in vitro that did not show signs of degeneration and did not investigate the parameter regurgitation between the surgical valve and THV as ViV. Therefore, our results may reflect differently from how the investigated THVs behave within degenerated bioprostheses in vivo. Further, we used 26 mm THVs and not the 23 mm THV, which is recommended for implantation inside 21 mm surgical valves. Additionally, we chose a 21 mm labelled size for all surgical valve models, which have different true internal diameters. Finally, our data only provide functional short-term results and do not provide information on long-term outcomes.

## 5. Conclusions

Our data demonstrate the hydrodynamic superiority of Evolut R over Evolut PRO in all the investigated SAVs, except inside the rapid deployment valve, where Evolut PRO was superior. Moreover, the SAV model significantly impacts the hydrodynamics of the THV. Therefore, in the clinical setting of a lifetime strategy for a respective patient, selecting the primary biological SAV should be performed carefully, and an optimal pre-condition for a future ViV procedure should be created. The findings of our study provide important information on the choice of THV and THV-in-SAV combinations in daily clinical practice to achieve the best possible long-term results for our patients.

## Data Availability

The data supporting this study’s findings are available from the corresponding author upon reasonable request.

## Supplementary Materials

The following supporting information can be downloaded at: https://www.mdpi.com/article/10.3390/jcdd11080244/s1, Table S1: Effective orifice area (EOA) and mean pressure gradient (MPG) of Evolut R in surgical aortic valves (SAV); Table S2: Effective orifice area (EOA) and mean pressure gradient (MPG) of Evolut PRO in surgical aortic valves (SAV); Table S3: Maximum geometric orifice area (GOA), minimal internal diameter (MID) and pin-wheeling index of Evolut R and Evolut PRO inside surgical aortic valves (SAV). Video S1: Evolut R and Evolut PRO as valve-in-valve in Hancock II, Mosaic Ultra and Epic Supra. Video S2: Evolut R and Evolut PRO as valve-in-valve in Trifecta, Perimount, Magna Ease, Avalus, Freestyle and Intuity.
