# Supplementary material for: Comparison of Two Generations of Self-Expandable Transcatheter Heart Valves in Nine Surgical Valves: An In Vitro Study
Source: J Cardiovasc Dev Dis. 2024 Aug 8;11(8):244. doi: 10.3390/jcdd11080244 (PMC11354675; doi:10.3390/jcdd11080244)
Supplement: Supplementary file 1 [file jcdd-11-00244-s001.zip › Table S1.pdf]

Table S1: Effective orifice area (EOA) and mean pressure gradient (MPG) of Evolut R in surgical aortic valves (SAV)

Evolut R in SAV	EOA [cm²] median (25th-75th percentile)	Pairwise-comparison (p value)							
		Hancock II	Mosaic Ultra	Epic Supra	Trifecta	Perimount	Magna Ease	Avalus	Freestyle
**Hancock II**	1.55 (1.50-1.61)								
**Mosaic Ultra**	1.58 (1.51-1.61)	0.989							
**Epic Supra**	1.60 (1.56-1.74)	0.037	0.095						
**Trifecta**	1.77 (1.64-1.86)	<0.001	<0.001	0.008					
**Perimount**	1.90 (1.74-2.01)	<0.001	<0.001	<0.001	0.041				
**Magna Ease**	1.92 (1.79-2.02)	<0.001	<0.001	<0.001	<0.001	0.368			
**Avalus**	1.95 (1.85-2.05)	<0.001	<0.001	<0.001	<0.001	0.238	0.746		
**Freestyle**	1.96 (1.85-2.04)	<0.001	<0.001	<0.001	<0.001	0.112	0.297	0.662	
**Intuity**	1.89 (1.78-1.98)	<0.001	<0.001	<0.001	0.009	0.831	0.505	0.365	0.143
	**MPG [mmHg] median (25th-75th percentile)**								
**Hancock II**	9.45 (8.18-11.09)								
**Mosaic Ultra**	9.53 (8.16-10.86)	0.968							
**Epic Supra**	7.8 (6.97-9.07)	0.089	0.063						
**Trifecta**	7.73 (6.71-8.69)	0.011	0.004	0.592					
**Perimount**	7.02 (6.19-7.94)	<0.001	<0.001	0.046	0.197				
**Magna Ease**	6.79 (5.93-7.58)	<0.001	<0.001	0.015	0.053	0.496			
**Avalus**	6.46 (5.75-7.27)	<0.001	<0.001	0.003	0.02	0.247	0.715		
**Freestyle**	6.24 (5.34-7.19)	<0.001	<0.001	<0.001	0.002	0.08	0.175	0.407	
**Intuity**	5.79 (5.15-7.06)	<0.001	<0.001	<0.001	0.005	0.08	0.232	0.407	0.951
