# Supplementary material for: Comparison of Two Generations of Self-Expandable Transcatheter Heart Valves in Nine Surgical Valves: An In Vitro Study
Source: J Cardiovasc Dev Dis. 2024 Aug 8;11(8):244. doi: 10.3390/jcdd11080244 (PMC11354675; doi:10.3390/jcdd11080244)
Supplement: Supplementary file 1 [file jcdd-11-00244-s001.zip › Table S2.pdf]

Table S2: Effective orifice area (EOA) and mean pressure gradient (MPG) of Evolut PRO in surgical aortic valves (SAV)

Evolut PRO in SAV	EOA [cm^2] median (25th-75th percentile)	Pairwise-comparison (p value)							
		Hancock II	Mosaic Ultra	Epic Supra	Trifecta	Perimount	Magna Ease	Avalus	Freestyle
**Hancock II**	1.53 (1.48-1.60)								
**Mosaic Ultra**	1.58 (1.51-1.67)	0.284							
**Epic Supra**	1.58 (1.54-1.62)	0.076	0.841						
**Trifecta**	1.60 (1.53-1.70)	0.023	0.512	0.367					
**Perimount**	1.79 (1.66-1.88)	<0.001	0.001	<0.001	0.003				
**Magna Ease**	1.79 (1.66-1.89)	<0.001	0.001	<0.001	0.004	1			
**Avalus**	1.78 (1.64-1.94)	<0.001	0.003	<0.001	0.005	0.736	0.736		
**Freestyle**	1.84 (1.72-1.94)	<0.001	0.002	<0.001	0.003	0.491	0.489	0.736	
**Intuity**	1.98 (1.93-2.18)	<0.001	<0.001	<0.001	<0.001	<0.001	<0.001	<0.001	0.003
	**MPG [mmHg] median (25th-75th percentile)**								
**Hancock II**	10.25 (8.63-11.94)								
**Mosaic Ultra**	9.47 (7.86-11.36)	0.571							
**Epic Supra**	8.57 (7.47-10.20)	0.074	0.326						
**Trifecta**	9.01 (7.73-10.88)	0.213	0.569	0.694					
**Perimount**	7.77 (6.62-8.80)	0.002	0.018	0.161	0.045				
**Magna Ease**	7.54 (6.61-8.88)	0.002	0.02	0.127	0.049	0.989			
**Avalus**	7.86 (6.99-8.94)	0.003	0.028	0.275	0.075	0.989	0.989		
**Freestyle**	7.36 (6.21-8.34)	0.002	0.013	0.082	0.02	0.471	0.473	0.451	
**Intuity**	4.99 (4.26-6.33)	<0.001	<0.001	<0.001	<0.001	<0.001	<0.001	<0.001	0.001
