# Supplementary material for: Comparison of Two Generations of Self-Expandable Transcatheter Heart Valves in Nine Surgical Valves: An In Vitro Study
Source: J Cardiovasc Dev Dis. 2024 Aug 8;11(8):244. doi: 10.3390/jcdd11080244 (PMC11354675; doi:10.3390/jcdd11080244)
Supplement: Supplementary file 1 [file jcdd-11-00244-s001.zip › Table S3.pdf]

Table S3: Maximum geometric orifice area (GOA), minimal internal diameter and pin-wheeling index of Evolut R and Evolut PRO inside surgical aortic valves (SAV)

Evolut R in SAV	max GEO [cm^2]	MID [mm]	Pin-wheeling index [%]			
			Leaflet 1	Leaflet 2	Leaflet 3	mean
**Hancock II**	1.1	12	29	29	24	27
**Mosaic Ultra**	1.3	13	21	23	29	24
**Epic Supra**	1.4	13	22	20	14	19
**Trifecta**	1.4	13	23	14	18	18
**Perimount**	1.5	14	24	14	9	16
**Magna Ease**	1.7	15	18	13	23	18
**Avalus**	1.4	13	10	23	18	17
**Freestyle**	1.7	15	22	11	10	14
**Intuity**	1.9	16	16	16	14	15
**Evolut PRO in SAV**						
**Hancock II**	1.0	11	23	17	22	21
**Mosaic Ultra**	1.5	14	7	33	14	18
**Epic Supra**	1.4	13	27	24	7	19
**Trifecta**	1.2	12	13	13	26	17
**Perimount**	1.3	13	7	20	21	16
**Magna Ease**	1.5	14	21	10	11	14
**Avalus**	1.4	13	21	14	10	15
**Freestyle**	1.7	15	22	5	15	14
**Intuity**	2.0	16	17	14	9	13
